# Mixed convection stagnation point flow of the blood based hybrid nanofluid around a rotating sphere

**DOI:** 10.1038/s41598-021-86868-x

**Published:** 2021-04-02

**Authors:** Taza Gul, Basit Ali, Wajdi Alghamdi, Saleem Nasir, Anwar Saeed, Poom Kumam, Safyan Mukhtar, Wiyada Kumam, Muhammad Jawad

**Affiliations:** 1 Department of Mathematics, City University of Science and IT, Peshawar, 25000 KP Pakistan; 2 Higher Education Department, Khyber Pakhtunkhwa, Peshawar, 25000 KP Pakistan; 3 Department of Information Technology, Faculty of Computing and Information Technology, King Abdulaziz University, Jeddah, 80261 Saudi Arabia; 4 Department of Mathematics, Abdul Wali Khan University, Mardan, 23200 KP Pakistan; 5 Center of Excellence in Theoretical and Computational Science (TaCS-CoE), Faculty of Science, King Mongkut’s University of Technology Thonburi (KMUTT), 126 Pracha Uthit Rd., Bang Mod, Thung Khru, Bangkok, 10140 Thailand; 6 Department of Medical Research, China Medical University Hospital, China Medical University, Taichung, 40402 Taiwan; 7 Basic Sciences Department, Deanship of Preparatory Year, King Faisal University, Al Ahsa, 31982 Saudi Arabia; 8 Program in Applied Statistics, Department of Mathematics and Computer Science, Faculty of Science and Technology, Rajamangala University of Technology Thanyaburi, Thanyaburi, 12110 Pathumthani Thailand

**Keywords:** Energy science and technology, Mathematics and computing, Nanoscience and technology, Physics

## Abstract

In this new world of fluid technologies, hybrid nanofluid has become a productive subject of research among scientists for its potential thermal features and abilities, which provides an excellent result as compared to nanofluids in growing the rate of heat transport. Our purpose here is to introduce the substantial influences of magnetic field on 2D, time-dependent and stagnation point inviscid flow of couple stress hybrid nanofluid around a rotating sphere with base fluid is pure blood, ${\text{TiO}}_{2} \,\,{\text{and}}\,\,{\text{Ag}}$ as the nanoparticles. To translate the governing system of partial differential equations and the boundary conditions relevant for computation, some suitable transformations are implemented. To obtain the analytical estimations for the corresponding system of differential expression, the innovative Optimal Homotopy Analysis Method is used. The characteristics of hybrid nanofluid flow patterns, including temperature, velocity and concentration profiles are simulated and analyzed in detail due to the variation in the evolving variables. Detailed research is also performed to investigate the influences of relevant constraints on the rates, momentum and heat transport for both ${\text{TiO}}_{2} + {\text{Ag}} + Blood$ and ${\text{TiO}}_{2} + Blood$. One of the many outcomes of this analysis, it is observed that increasing the magnetic factor will decelerate the hybrid nanofluid flow velocity and improve the temperature profile. It may also be demonstrated that by increasing the Brownian motion factor, significant improvement can be made in the concentration field of hybrid nanofluid. The increase in the nanoparticle volume fraction from 0.01 to 0.02 in the case of the hybrid nanofluid enhances the thermal conductivity from 5.8 to 11.947% and for the same value of the nanoparticle volume fraction in the case of nanofluid enhance the thermal conductivity from 2.576 to 5.197%.

## Introduction

In recent studies, bio-nanotechnology is one of the revolutionary approaches that unlock the new frontiers in the field of biological science, medicine and engineering industries. This development involves producing and studying tiny materials supposed to be 1 nm to 100 nm in dimension. The synthesis of nanomaterials and natural sciences aims to introduce different novel nano-devices that allow revolutionary phenomena and biomedical discoveries to be explored at the molecular level. Such advancements provide science with various techniques and instruments for clinical testing, medicinal and preventative healthcare applications. For that cause, an extension of certain nano-devices, nanomaterials, including their implementations in biotechnology, drugs, and engineering industries, is explored by many other investigators^1–3^. In 1904, Maxwell^4^ specifically initiated the theory of retaining micro-scale particles in coolants, so it did not gain significant interest due to any drawbacks. Anyway, the theory once again captured the interest of researchers following the invention of nanoparticles. Choi^5^ initially interpreted the expression Nanofluid. Sheikholeslami^6^ simply stated a nanofluid is just a liquid comprising tiny sized elements known as nanoparticles. In general, such nanoparticles are made of ceramic $\left( {{\text{Al}}_{2} {\text{O}}_{3} ,\;{\text{CuO}}} \right)$, metals $({\text{Ag}},\,{\text{Cu}},\,{\text{Au}})$, metal nitrides $\left( {{\text{AlN}},\;{\text{SiN}}} \right)$, Ferro particles $\left( {{\text{CoFe}}_{2} {\text{O}}_{4} ,\,{\text{Fe}}_{3} {\text{O}}_{4} ,\,{\text{Mn - ZnFe}}_{2} {\text{O}}_{4} } \right)$, carbon in various forms (diamonds, graphite and carbon nanotubes) etc. Parvin and Chamkha^7^ documented the heat transport, convection fluid flow and entropy generation in longitudinal and transverse enclosure form. Cu/water nanofluid occupies the space. Specifically, the quantitative simulation explains the influence of the fluid flow factors on Bejan and Nusselt numbers. A comprehensive analysis of the Buongiorno nanofluid model for the solution of natural convectional flow in a partly warmed curly space fill utilizing nanofluid has been performed by Pop et al.^8^. In another investigation, Ghasemian et al.^9^ examined the Buongiorno model to analyze the 3D uncontrolled nanofluid movement through a square tube with a sinusoidal diameter. The concentration has been focused on scientific research, analysis involving the saturation of two or even more nanomaterials in common fluid, termed hybrid nanofluid/ compound fluid^10^.

Many scholars have made some important attempts to investigate these types of hybrid nanofluid flows and many reports are available that cast the light on the possible features of those sorts of frameworks. In specific, nanofluid is quite recognized as a high heat transport fluid. In this article, the hybrid nanofluid is discussed further to improve the conventional nanofluid's heat transport efficiency. Recently, various numerical studies were examined on hybrid nanofluids as a new idea in science and technology. Devi and Devi^11^ scrutinized the problems of heat transfer and flow of hydro-magnetic hybrid nanofluids $\left( {{\text{Cu - Al}}_{2} {\text{O}}_{3} /{\text{water}}} \right)$ through an extending surface. Tayebi and Chamkha^12^ computed numerically the problem of heat transport of hybrid nanofluids $\left( {{\text{Cu - Al}}_{2} {\text{O}}_{3} /{\text{water}}} \right)$ in an annulus. In another investigation, the characteristics of ${\text{TiO}}_{2} {\text{ - Cu}}/{\text{H}}_{2} {\text{O}}$ hybrid nanofluid with Lorentz force were scrutinized by Ghadikolaei et al.^13^. Hayat et al.^14^ inspected the rotating flow problem of ${\text{Ag - CuO}}/{\text{water}}$ hybrid nanofluids. The aqueous Titania-copper hybrid nanofluid stagnation point flow towards the stretching tube was explored by Yousefi et al.^15^. Subhani and Nadeem^16^ studied the behavior of ${\text{Cu - TiO}}_{2} /{\text{H}}_{2} {\text{O}}$(hybrid nanofluid) over the stretching surface. Dinarvand et al.^17^ introduced ${\text{CuO - Cu}}$/blood (hybrid nanofluid) circulation on a permeable stretching sheet, which is an advanced density concept, that can be a favorable model in medical sciences, particularly in cancer therapy and drug delivery. In an investigation for cancer cell therapy, Liu et al.^18^ examined that ${\text{Pt - TiO}}_{2}$ and ${\text{Au - TiO}}_{2}$ are the best nanocomposites. Through their laboratory analysis they found in the involvement of ${\text{TiO}}_{2}$ or ${\text{Au - TiO}}_{2}$ nanoparticles, after two hours of treatment with UV irradiation the remaining fraction of cancer cell was diminished in both situations. In summary, this has been reported that most cancer cells will destroy using metal-${\text{TiO}}_{2}$ nanocomposite particles than ${\text{TiO}}_{2}$ nanoparticles single which illustrates the need to use nano-materials in medicines. In combination with its special characteristics, ${\text{Ag}}$ (silver) has various biomedical uses, such as permeability, strength, electrochemical and anti-bacterial properties. For anti-bacterial activities against the slew of micro-organisms such as microbes, protozoa, fungi, even the latest viruses, ${\text{Ag}}$ and ${\text{Ag}}$-related substances are used. Their contributions show the anti-tumor effect of ${\text{Ag}}$ particles which indicate that they could be a cost-effective cancer therapy then another treatment^19^. Some recent studies regarding hybrid nanofluids flows with different geometries are investigated by Zainal et al.^20–22^ and Dinarvand^23–25^.

In different engineering fields, the fluid flowing around hot rotating frames has many implementations, including gravitational chemical processing, advanced technology, spin-stabilized rocket temperature control, electric pumps and generators, thermal plasma manufacturing, and painting spray processes. Takhar and Nath^26^ have investigated the self-similar approach for passing flow at the stagnation point through a spinning sphere in the presence of magnetic force influences. In another work, Anilkumar and Roy^27^ scrutinized time-dependent variable viscosity movement in the zone of stagnation point a spinning sphere under which the free-stream momentum and angular speed of the revolving sphere very gradually with time. The variational magnetic, thermal convectional flow via a spinning cone in the transversely isotropic porous surface was investigated by Beg et al.^28^. Chamkha and Ahmad^29^ calculated the numerical result of time-dependent MHD free and mixed convectional motion and heat transport via a spinning sphere. More recently, Mahdy et al.^30^ introduced a mathematical model to determine the Casson fluid flow of due to rotating sphere in the presence of conjugate MHD, entropy generation and convective boundary conditions.

Although the base fluid is “pure blood” in our current model, it must be remembered that the blood viscidness is not really consistent from the medical perspective, and it will be changed not only through vessel diameter but also by temperature hematocrit factor and stress. In numerous investigations, this has been also specified that Newtonian blood performance's presumption is suitable for high shear rate motion, for instance, inflow across blood vessels. Usually, as the blood circulation improves, the blood thickness decreases, improving the speed of blood flow and reducing clotting factors^31^. The curved flexible artery through which the blood's viscosity is presumed to be temperature-dependent was studied by Akbar and Nadeem^32^. The statistical blood flow model with circular-based viscosity was examined by Gupta et al.^33^. Ijaz et al.^34^ analyzed the theoretical results of hemodynamics with unique features utilizing ${\text{Cu - CuO}}$/blood, hybrid nanofluid flow in stenosized arteries. Ellahi et al.^35^ conducted a numerical investigation of the peristaltic flow of a couple stress-based gold nanofluid among the hole of dual co-axial cylinders with various outlines and structures. Chahregh and Dinarvand^36^ examined the circulation of ${\text{TiO}}_{2} {\text{ - Ag}}$/blood, hybrid nanofluid across a vessel for blood as well as drug transport applications in the respiration process.

Also, to the best of the authors' knowledge in the relevant literature, no one has ever tried to investigate the movement around a rotating sphere using pure blood as a based liquid and hybrid nano-materials to explain many real-life biomedical applications and medication filed and transportation. Supported by the said research findings, this article analyzes an appropriate framework of couple stress hybrid nanofluid movement of mixed convection across a spinning sphere in the immersion of MHD, thermophoresis and Brownian motion effect. The hybrid nanofluid is illustrated by maintaining two separate Titania $\left( {{\text{TiO}}_{2} } \right)$ and silver $\left( {{\text{Ag}}} \right)$ nanoparticles in the base fluid blood. The governing equations including constraints are transformed into a boundary value problem of differential equations based on the similarity transformation. Using the OHAM (Optimal Homotopy Analysis Method) in Mathematica, the system of equations is then explained analytically. Moreover, the consequences of several other variables' characteristics of flow and heat transport are seen numerically and graphically.

## Mathematical formulation

**Figure 1:**
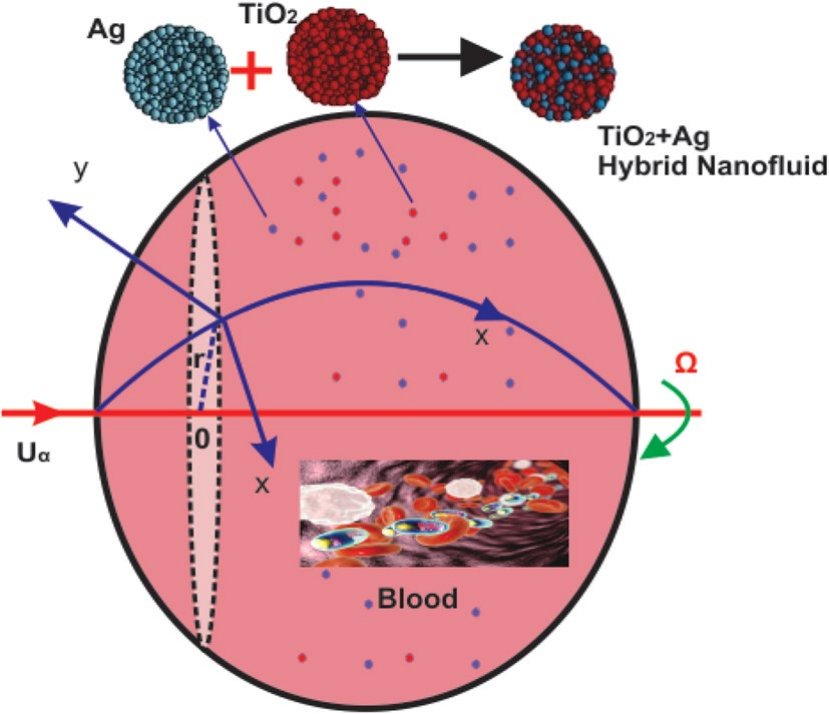
Configuration of model problem.

In the stagnation point zone around a rotating sphere, we assume a time-dependent flow of mixed convection of the couple stress hybrid nanofluid ${\text{TiO}}_{2} {\text{ - Ag}}$/blood with the Buongiorno's mathematical model flow along with convective boundary conditions. Figure 1 demonstrates the working model and related coordinate structure of the problem. The model is totally based on the theoretical analysis and the experimental data in Table 3 is used from the existing literature reference^36^. The working problem is presented on some specific assumptions:

i. The x-axis is determined on the surface of a sphere and the y-axis is normal to it.
ii. The magnetic field $B(t) = B_{0} t^{{\frac{ - 1}{2}}}$ is considered perpendicular to the flow field.
iii. It is supposed that the temperature and concentration at the surface of the sphere have $T_{w} ,\,C_{w}$ where $T_{\infty } ,\,C_{\infty }$ the ambient temperature and concentration.
iv. The viscus dissipation terms are negligible.
v. Many mechanisms, including thermophoresis, Brownian motion and the MHD impacts are taken into consideration.
vi. The nanoparticles are supposed to be in thermal equilibrium.

In view of such flow assumptions, the resulting equations interpreted as^30,36^:

1 $$ \frac{{\partial \left( {ru} \right)}}{\partial x} + \frac{{\partial \left( {rv} \right)}}{\partial y} = 0 $$

2 $$ \begin{aligned} & \frac{\partial u}{{\partial t}} + u\frac{\partial u}{{\partial x}} + v\frac{\partial u}{{\partial y}} - \left( {\frac{{w^{2} }}{r}} \right)\frac{dr}{{dx}} = \frac{\partial U}{{\partial t}} + U\frac{\partial U}{{\partial x}} + v_{hnf} \frac{{\partial^{2} u}}{{\partial y^{2} }} - \frac{{\sigma_{hnf} B_{0}^{2} }}{{\rho_{hnf} }}\left( {u - U} \right) \\ & \quad - \,\frac{{\eta_{0} }}{{\rho_{hnf} }}\frac{{\partial^{4} u}}{{\partial y^{4} }} + \frac{{g\beta_{T}^{*} \rho_{f\infty } }}{{\rho_{hnf} }}\left( {T - T_{\infty } } \right) - \frac{{g\beta_{C}^{*} \rho_{f\infty } }}{{\rho_{hnf} }}\left( {C - C_{\infty } } \right), \\ \end{aligned} $$

3 $$ \frac{\partial w}{{\partial t}} + u\frac{\partial w}{{\partial x}} + v\frac{\partial w}{{\partial y}} + \left( \frac{wu}{r} \right)\frac{dr}{{dx}} = v_{hnf} \frac{{\partial^{2} w}}{{\partial y^{2} }} - \frac{{\sigma_{hnf} B_{0}^{2} w}}{{\rho_{hnf} }} - \frac{{\eta_{0} }}{{\rho_{hnf} }}\frac{{\partial^{4} w}}{{\partial y^{4} }}, $$

4 $$ \frac{\partial T}{{\partial t}} + u\frac{\partial T}{{\partial x}} + v\frac{\partial T}{{\partial y}} = \alpha_{hnf} \frac{{\partial^{2} T}}{{\partial y^{2} }} + \tau \left( {D_{B} \frac{\partial T}{{\partial y}}\frac{\partial C}{{\partial y}} + \frac{{D_{T} }}{{T_{\infty } }}\left( {\frac{\partial T}{{\partial y}}} \right)^{2} } \right), $$

5 $$ \frac{\partial C}{{\partial t}} + u\frac{\partial C}{{\partial x}} + v\frac{\partial C}{{\partial y}} = D_{B} \frac{{\partial^{2} C}}{{\partial y^{2} }} + \frac{{D_{T} }}{{T_{\infty } }}\frac{{\partial^{2} T}}{{\partial y^{2} }}. $$

In said expressions $\tau = \frac{{\left( {\rho c_{p} } \right)_{hnf} }}{{\left( {\rho c_{p} } \right)_{f} }}$ heat capacity ratio with $\left( {\rho c_{p} } \right)_{f}$ is the blood heat capacity, $\left( {\rho c_{p} } \right)_{hnf}$ is the solid nanoparticles heat capacity, $\left( {D_{B} ,D_{T} } \right)$ are the diffusion coefficients of (Brownian, thermophoresis), $B_{0}$ is the magnetic field, $g$ is the gravitational acceleration,$u,\,v,w$ are velocities element in $x,y$ and $z$ directions, $\eta_{0}$ is the couple stress parameter, $\mu_{hnf}$, $\rho_{hnf}$, $\sigma_{{{\text{hnf}}}}$, $k_{hnf}$ and $\left( {\rho C_{p} } \right)_{hnf}$ are viscosity, density, electrical and thermal conductivities and specific heat, where hybrid nanofluid refer $hnf$. All such relations^11^ and defined in Tables 1 and 2.

**Table 1:** Thermo-physical traits of ${\text{TiO}}_{2}$**/**blood nanofluid^11,36^.

Viscosity	$\mu_{nf} = {{\mu_{f} } \mathord{\left/ {\vphantom {{\mu_{f} } {\left( {1 - \phi_{1} } \right)^{2.5} }}} \right. \kern-\nulldelimiterspace} {\left( {1 - \phi_{1} } \right)^{2.5} }}$
Density	$\rho_{nf} = \rho_{f} \left\{ {\left( {1 - \phi_{1} } \right) \, + \phi_{1} \, {{\rho_{{S_{1} }} } \mathord{\left/ {\vphantom {{\rho_{{S_{1} }} } {\rho_{f} }}} \right. \kern-\nulldelimiterspace} {\rho_{f} }}} \right\}$
Specific heat	${{\left( {\rho \, c_{p} } \right)_{nf} } \mathord{\left/ {\vphantom {{\left( {\rho \, c_{p} } \right)_{nf} } {\left( {\rho \, c_{p} } \right)_{f} }}} \right. \kern-\nulldelimiterspace} {\left( {\rho \, c_{p} } \right)_{f} }} = \left[ {\left( {1 - \phi_{1} } \right) + \phi_{1} \, \left( {{{\left( {\rho \, c_{p} } \right){}_{{S_{1} }}} \mathord{\left/ {\vphantom {{\left( {\rho \, c_{p} } \right){}_{{S_{1} }}} {\left( {\rho \, c_{p} } \right)_{f} }}} \right. \kern-\nulldelimiterspace} {\left( {\rho \, c_{p} } \right)_{f} }}} \right)} \right]$
Thermal conductivity	${{k_{nf} } \mathord{\left/ {\vphantom {{k_{nf} } {k_{f} }}} \right. \kern-\nulldelimiterspace} {k_{f} }} \, = \left( {k_{{_{{S_{1} }} }} + 2k_{f} - 2\phi_{1} \left( {k_{f} - k_{{S_{1} }} } \right)} \right)\left( {k_{{_{{S_{1} }} }} + 2k_{f} + 2\phi_{1} \left( {k_{f} - k_{{S_{1} }} } \right)} \right)^{ - 1}$

**Table 2:** Thermo-physical traits of ${\text{TiO}}_{2} + {\text{Ag}}/{\text{blood}}$^11,36^.

Viscosity	$\mu_{hnf} = {{\mu_{f} } \mathord{\left/ {\vphantom {{\mu_{f} } {\left( {1 - \phi_{1} } \right)^{2.5} \left( {1 - \phi_{2} } \right)^{2.5} }}} \right. \kern-\nulldelimiterspace} {\left( {1 - \phi_{1} } \right)^{2.5} \left( {1 - \phi_{2} } \right)^{2.5} }}$
Density	$\rho_{hnf} = \left( {1 - \phi_{2} } \right)\,\,\,\left\{ {\phi_{1} \, \rho_{{S_{1} }} + \,\,\left( {1 - \phi_{1} } \right) \, \,\rho_{f} } \right\} + \phi_{2} \, \rho_{{S_{2} }}$
Specific heat	${{\left( {\rho \, c_{p} } \right)_{hnf} } \mathord{\left/ {\vphantom {{\left( {\rho \, c_{p} } \right)_{hnf} } {\left( {\rho \, c_{p} } \right)_{f} }}} \right. \kern-\nulldelimiterspace} {\left( {\rho \, c_{p} } \right)_{f} }} = \left( {1 - \phi_{2} } \right) \cdot \left( {1 - \phi_{1} } \right) + \phi_{1} \cdot {{\left( {\rho \, c_{p} } \right){}_{{S_{1} }}} \mathord{\left/ {\vphantom {{\left( {\rho \, c_{p} } \right){}_{{S_{1} }}} {\left( {\rho \, c_{p} } \right)_{f} }}} \right. \kern-\nulldelimiterspace} {\left( {\rho \, c_{p} } \right)_{f} }} + \phi_{2} \, \cdot {{\left( {\rho \, c_{p} } \right){}_{{S_{2} }}} \mathord{\left/ {\vphantom {{\left( {\rho \, c_{p} } \right){}_{{S_{2} }}} {\left( {\rho \, c_{p} } \right)_{f} }}} \right. \kern-\nulldelimiterspace} {\left( {\rho \, c_{p} } \right)_{f} }}$
Thermal conductivity	${{k_{nf} } \mathord{\left/ {\vphantom {{k_{nf} } {k_{f} }}} \right. \kern-\nulldelimiterspace} {k_{f} }} \, = \left\{ {\frac{{2k_{nf} + k_{{S_{1} }} - 2\phi_{2} \cdot \left( {k_{nf} - k_{{S_{1} }} } \right)}}{{2k_{nf} + k_{{S_{1} }} + 2\phi_{2} \cdot \left( {k_{nf} - k_{{S_{1} }} } \right)}}} \right\} \cdot \left\{ {\frac{{2k_{f} + k_{{S_{2} }} - 2\phi_{1} \cdot \left( {k_{f} - k_{{S_{2} }} } \right)}}{{2k_{f} + k_{{S_{2} }} - 2\phi_{1} \cdot \left( {k_{f} - k_{{S_{2} }} } \right)}}} \right\}$

Initially, as the first nanoparticle we scattered ${\text{TiO}}_{2}$ (Titania) into the blood (base fluid) to produce a ${\text{TiO}}_{2} /{\text{blood}}$(mono-nano liquid). In our current ${\text{TiO}}_{2} /{\text{blood}}$(base liquid), ${\text{Ag}}$ (Silver) is then dispersed as a supplementary nanoparticle to construct the appropriate ${\text{TiO}}_{2} + {\text{Ag}}/{\text{blood}}$ (hybrid nanofluid). In this case, the subscript $S_{1}$ denotes ${\text{TiO}}_{2}$ (Titania nanoparticles), although subscript $S_{2}$ denotes to ${\text{Ag}}$ (Silver nanoparticles) and subscript $f$ mentions pure blood (base fluid). In Tables 1 and 2, $\phi_{1}$ and $\phi_{2}$ are refer the volume fraction of ${\text{TiO}}_{2}$ and ${\text{Ag}}$ nanoparticles, where $\phi_{1} = \phi_{2} = 0$ refer normal fluid.

### Initial and boundary conditions

Subject to the corresponding initial and boundary condition^30^

6 $$ \begin{aligned} & {\text{for}}\,\,t < 0,\,\,u\left( {t,x,y} \right) = 0,\;v\left( {t,x,y} \right) = 0,\,\,w\left( {t,x,y} \right) = 0,\;T\left( {t,x,y} \right) \to T_{\infty } ,\;C\left( {t,x,y} \right) \to C_{\infty } \\ & \quad y = 0:\,\,u\left( {t,x,y} \right) = 0,\,\,v\left( {t,x,y} \right) = 0,\,\,w\left( {t,x,y} \right) = \Omega \left( t \right)r \\ & {\text{for}}\,\,t \ge 0:\,\, - \frac{k}{h}\left( {\frac{\partial T}{{\partial y}}} \right) = \left( {T_{f} - T} \right),\,\,D\left( {\frac{\partial C}{{\partial y}}} \right) + \frac{D}{{T_{\infty } }}\left( {\frac{\partial T}{{\partial y}}} \right) = 0, \\ & \quad u\left( {t,x,\infty } \right) \to 0,\,\,U\left( {t,x,\infty } \right) \to 0,\,\,T\left( {t,x,\infty } \right) \to T_{\infty } ,\,\,C\left( {t,x,\infty } \right) \to C_{\infty } . \\ \end{aligned} $$

### Similarity variables

To renovate above expressions which represent the flow problem to dimensionless system, we use the following similarity transformation^30^

7 $$ \begin{aligned} \eta & = \left( {\frac{2}{{v_{f} \,t}}} \right)y,u = \left( {\frac{A\,x}{t}} \right)F^{\prime}\left( \eta \right),U\left( {t,x} \right) = \frac{A\,x}{t},w = \left( {\frac{B\,x}{t}} \right)G\left( \eta \right),\Omega \left( t \right) = \frac{B}{t} \\ v & = - \left( {\frac{{2v_{f} }}{t}} \right)AF\left( \eta \right),\,\,\theta \left( \eta \right) = \frac{{T - T_{\infty } }}{{T_{w} - T_{\infty } }},\,\,\Phi \left( \eta \right) = \frac{{C - C_{\infty } }}{{C_{w} - C_{\infty } }} \\ \end{aligned} $$

Invoking the similarity factors in Eq. (7) in model Eqs. (1)–(5) with boundary condition Eq. (1) converted into the following form:

8 $$ \begin{aligned} & F^{\prime\prime\prime} + \left( {1 - \phi_{1} } \right)^{2.5} \left( {\left( {1 - \phi_{2} } \right)^{2.5} } \right)\left[ {\left( {1 - \phi_{2} } \right)\left\{ {1 - \left( {1 - \frac{{\rho_{{S_{1} }} }}{{\rho_{f} }}} \right)\phi_{1} } \right\} + \phi_{2} \frac{{\rho_{{S_{2} }} }}{{\rho_{f} }}} \right]A\left[ \begin{gathered} FF^{\prime\prime} - \frac{1}{2}\left( {1 - F - \frac{1}{2}\eta \,F^{\prime\prime}} \right) + \hfill \\ \frac{A}{2}\left( {1 - (F^{\prime})^{2} + \lambda G^{2} } \right) \hfill \\ \end{gathered} \right] \\ & \quad + \left( {1 - \phi_{1} } \right)^{2.5} \left( {1 - \phi_{2} } \right)^{2.5} \left[ {\frac{A}{2}\lambda^{*} \left( {\theta - {\text{Nr}}\,\Phi } \right) - \frac{1}{2}M\left( {F^{\prime} - 1} \right)} \right] - k^{*} F^{v} = 0, \\ \end{aligned} $$

9 $$ \begin{aligned} & G^{\prime\prime} + \left( {1 - \phi_{1} } \right)^{2.5} \left( {\left( {1 - \phi_{2} } \right)^{2.5} } \right)\left[ {\left( {1 - \phi_{2} } \right)\left\{ {1 - \left( {1 - \frac{{\rho_{{S_{1} }} }}{{\rho_{f} }}} \right)\phi_{1} } \right\} + \phi_{2} \frac{{\rho_{{S_{2} }} }}{{\rho_{f} }}} \right]\left[ {A\left( {FG^{\prime} - F^{\prime}\,G} \right) + \frac{1}{2}\left( {G + \frac{1}{2}\eta \,G^{\prime}} \right)} \right] \\ & \quad - \frac{{\left( {1 - \phi_{1} } \right)^{2.5} \left( {\left( {1 - \phi_{2} } \right)^{2.5} } \right)}}{2}MG - k^{*} G^{iv} = 0, \\ \end{aligned} $$

10 $$ \frac{{k_{hnf} }}{{k_{f} }}\theta^{\prime\prime} + \left[ {\left( {1 - \phi_{2} } \right)\left\{ {1 - \left( {1 - \frac{{\left( {\rho C_{p} } \right)_{{S_{1} }} }}{{\left( {\rho C_{p} } \right)_{f} }}} \right)\phi_{1} } \right\} + \phi_{2} \frac{{\left( {\rho C_{p} } \right)_{{S_{2} }} }}{{\left( {\rho C_{p} } \right)_{f} }}} \right]\Pr \left( {A\,F\,\theta^{\prime} + \frac{1}{4}\eta \,\,\theta^{\prime} + N_{b} \theta^{\prime}\,\,\Phi^{\prime} + N_{t} (\theta^{\prime})^{2} } \right) = 0, $$

11 $$ \left( {1 - \phi_{1} } \right)\left( {1 - \phi_{2} } \right)\Phi^{\prime\prime} + A{\text{Sc}}f\Phi^{\prime} + \frac{1}{4}\eta {\text{Sc}}\Phi + \frac{{N_{t} }}{{N_{b} }}\theta^{\prime\prime} = 0. $$

Initial and boundary conditions in Eq. (6) transform into the following form

12 $$ \begin{aligned} & F\left( 0 \right) = F^{\prime}\left( 0 \right) = 0\,\,\,,G\left( 0 \right) = 1,\,\,\,\,\theta^{\prime}\left( 0 \right) = B_{i} \left( {\theta \left( 0 \right) - 1} \right), \\ & N_{b} \Phi^{\prime}\left( 0 \right) + N_{t} \theta^{\prime}\left( 0 \right) = 0,\,\,F^{\prime}\left( \infty \right) \to 1,\,\,G\left( \infty \right) = \theta \left( \infty \right) = \Phi \left( \infty \right) \to 0. \\ \end{aligned} $$

Here the Schmidt number, Prandtl number, Reynolds number, Couple stress parameter, Biot number, Grashof number, Buoyancy ratio, Brownian motion and thermophoresis parameters, the rotation parameter, mixed convection parameter, and magnetic field parameter are

13 $$ \begin{aligned}  {\text{Sc}} = \frac{{v_{f} }}{{D_{B} }},\Pr = \frac{{\mu_{f} c_{p} }}{{k_{f} }},{\text{Re}} = \frac{Ux}{{v_{f} }},\,k^{*} = \frac{{2\eta_{0}t }}{{\rho_{f} \upsilon_{f}^{2} }}, {\text{Gr}} = \frac{{g\beta_{T}^{*} \left( {T_{w} - T_{\infty } } \right) \left( {C_{w} - C_{\infty } } \right)\rho f_{\infty } x^{3} }}{{v_{f}^{2} }}, \\  {\text{Nr}} = \frac{{(\rho_{p }-\rho_{f }) C_{\infty } }}{{\rho _{\infty } \beta_{C}^{*} \left( {C_{w} - C_{\infty } } \right)\left( {T_{w} - T_{\infty } } \right)}},N_{b} = \tau \frac{{D_{B} ({C_{w} - C_{\infty } }) }}{{v_{f} }},N_{t} = \tau \frac{{D_{T} \left( {T_{w} - T_{\infty } } \right)}}{{v_{f} T_{\infty } }},\lambda = \left( \frac{B}{A} \right)^{2} ,\lambda^{ * } = \frac{{{\text{Gr}}}}{{{\text{Re}}^{2} }},M = \frac{{\sigma B_{0}^{2} t}}{{\rho_{f} }}. \\ \end{aligned} $$

### Skin friction and Nusselt number

14 $$ \begin{aligned} & {\text{Re}}^{\frac{1}{2}} A^{\frac{1}{2}} C_{fx} = \frac{{2\mu_{hnf} }}{{\rho_{f} U^{2} }}\left( {\frac{\partial u}{{\partial y}}} \right)_{y = 0} = \frac{2\sqrt 2 }{{\left( {1 - \phi_{1} } \right)^{2.5} \left( {1 - \phi_{2} } \right)^{2.5} }}F^{\prime\prime}(0), \\ & {\text{Re}}^{\frac{1}{2}} A^{\frac{1}{2}} C_{fz} = \frac{{2\mu_{hnf} }}{{\rho_{f} U^{2} }}\left( {\frac{\partial w}{{\partial y}}} \right)_{y = 0} = \frac{ - 2\sqrt 2 \lambda }{{\left( {1 - \phi_{1} } \right)^{2.5} \left( {1 - \phi_{2} } \right)^{2.5} }}G^{\prime}(0), \\ & {\text{Re}}^{{\frac{ - 1}{2}}} A^{\frac{1}{2}} {\text{Nu}} = \frac{{ - k_{hnf} }}{{k_{f} (T_{w} - T_{\infty } )}}\left( {\frac{\partial T}{{\partial y}}} \right)_{y = 0} = \frac{{ - 2k_{hnf} }}{{k_{f} }}\theta^{\prime}(0). \\ \end{aligned} $$

## Solution methodology

The newly introduced BVPh 2.0 package of OHAM^37,38^ has been used to find the solution of the nonlinear problem. This package has the tendency to obtain the outputs of the modeled problem in short time. The recent solution has been achieved using the above-mentioned package. The trail solution for the modeled problem is obtained as:

15 $$ \begin{gathered} F_{0} \left( \eta \right) = \eta - \eta e^{ - \eta } ,G_{0} \left( \eta \right) = e^{ - \eta } , \hfill \\ \theta_{0} \left( \eta \right) = \frac{{B_{i} }}{{1 + B_{i} }}e^{ - \eta } ,\Phi_{0} \left( \eta \right) = - \frac{{N_{t} }}{{N_{b} }}\left( {\frac{{B_{i} }}{{1 + B_{i} }}} \right)e^{ - \eta } . \hfill \\ \end{gathered} $$

The square residual error for each one Eqs. (9–11) is obtained through:

16 $$ \varepsilon_{k}^{F} (h_{F} ,h_{\theta } ,h_{\Phi } ) = \frac{1}{L + 1}\sum\limits_{J = 0}^{L} {\left[ {\sum\limits_{m = 0}^{k} {(F_{m} )_{\eta } = j\pi \eta ,\sum\limits_{m = 0}^{k} {(\theta_{m} )_{\eta } = j\pi \eta ,\,\sum\limits_{m = 0}^{k} {(\Phi_{m} )_{\eta } = j\pi \eta \,} \,} } } \right]}^{2} \,, $$

17 $$ \varepsilon_{k}^{G} (h_{F} ,h_{G} ) = \frac{1}{L + 1}\sum\limits_{J = 0}^{L} {\left[ {\sum\limits_{m = 0}^{k} {(G_{m} )_{\eta } = j\pi \eta \,,\,\sum\limits_{m = 0}^{k} {(F_{m} )_{\eta } = j\pi \eta \,} \,} } \right]}^{2} \,, $$

18 $$ \varepsilon_{k}^{\theta } (h_{\theta } ,h_{F} ,h_{\Phi } ) = \frac{1}{L + 1}\sum\limits_{J = 0}^{L} {\left[ {\sum\limits_{m = 0}^{k} {(\theta_{m} )_{\eta } = j\pi \eta ,\sum\limits_{m = 0}^{k} {(F_{m} )_{\eta } = j\pi \eta ,\,\sum\limits_{m = 0}^{k} {(\Phi_{m} )_{\eta } = j\pi \eta \,} \,} } } \right]}^{2} \,, $$

19 $$ \varepsilon_{k}^{\Phi } (h_{\Phi } ,h_{\theta } ,h_{F} ) = \frac{1}{L + 1}\sum\limits_{J = 0}^{L} {\left[ {\sum\limits_{m = 0}^{k} {(\Phi_{m} )_{\eta } = j\pi \eta ,\sum\limits_{m = 0}^{k} {(F_{m} )_{\eta } = j\pi \eta ,\,\sum\limits_{m = 0}^{k} {(\theta_{m} )_{\eta } = j\pi \eta \,} \,} } } \right]}^{2} \,, $$

20 $$ \varepsilon^{t} = \varepsilon_{k}^{F} + \varepsilon_{k}^{G} + \varepsilon_{k}^{\theta } + \varepsilon_{k}^{\Phi } . $$

The sum of the total residual errors $\varepsilon_{m}^{t}$ has been attained from the velocity, temperature and concentration profiles.

## Result and discussion

**Figure 2:**
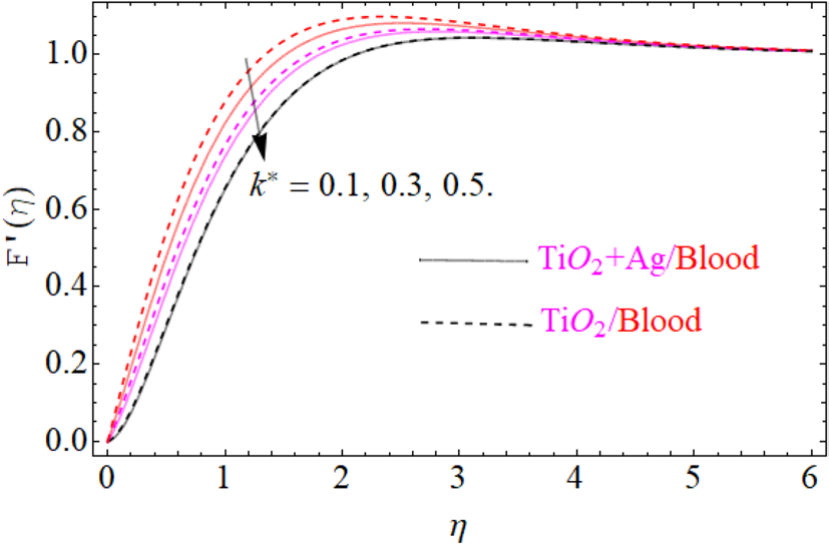
Under the influence of $k^{*}$ (couple stress parameter), $F^{\prime}\left( \eta \right)$.

**Figure 3:**
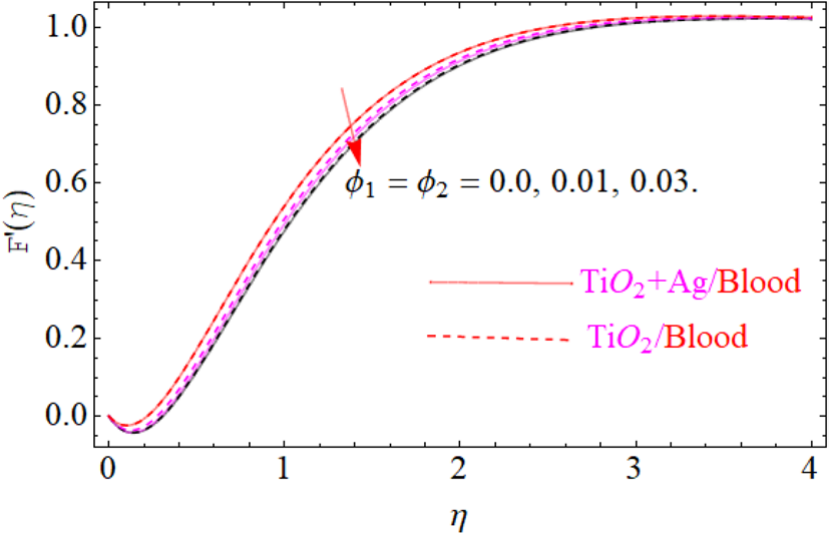
Under the influence of $\phi_{1} ,\phi_{2}$ (Nanoparticle volume fraction), $F^{\prime}\left( \eta \right)$.

**Figure 4:**
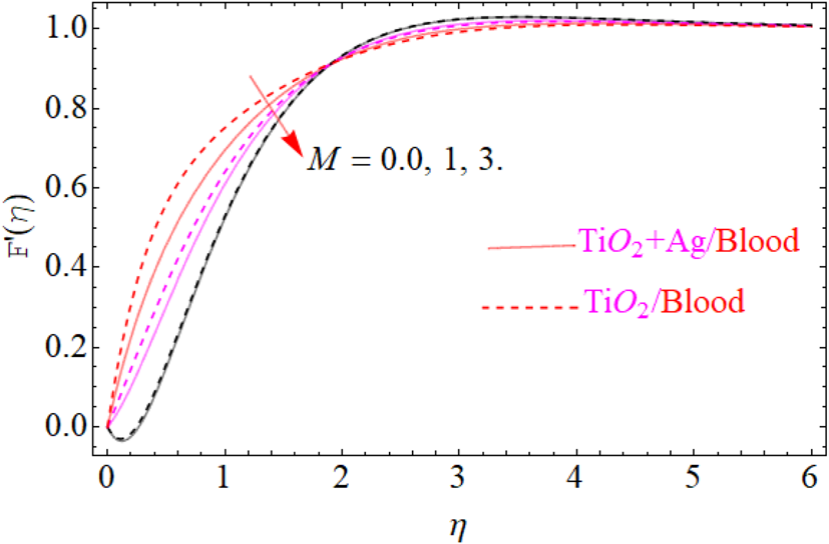
Under the influence of $M$ (Magnetic field parameter), $F^{\prime}\left( \eta \right)$.

**Figure 5:**
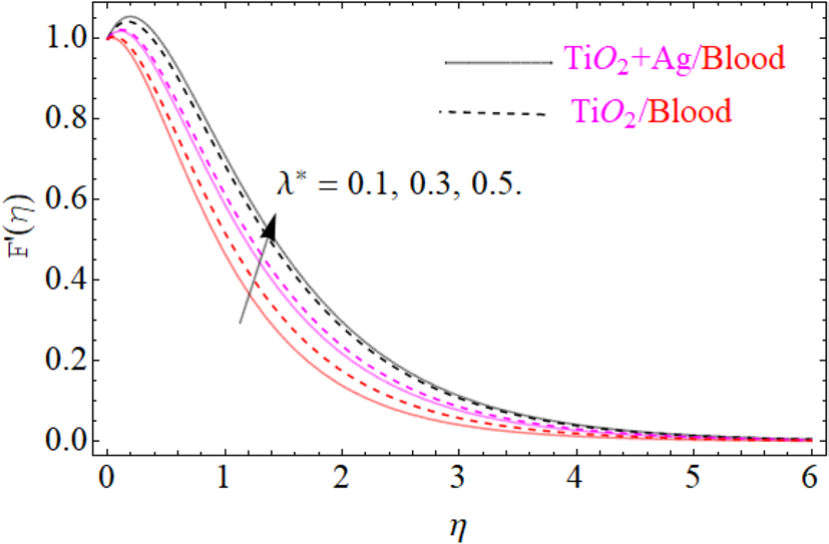
Under the influence of $\lambda^{*}$ (mixed convection parameter), $F^{\prime}\left( \eta \right)$.

**Figure 6:**
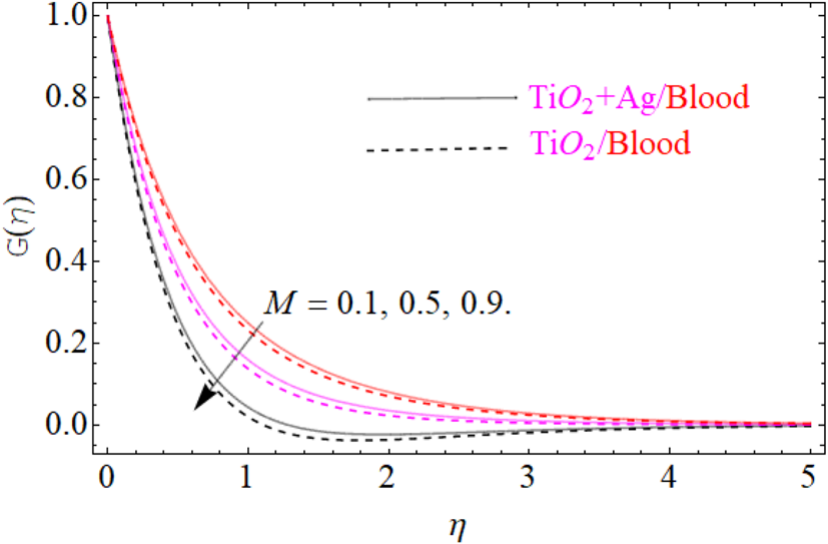
Under the influence of $M$ (Magnetic parameter), $G\left( \eta \right)$.

**Figure 7:**
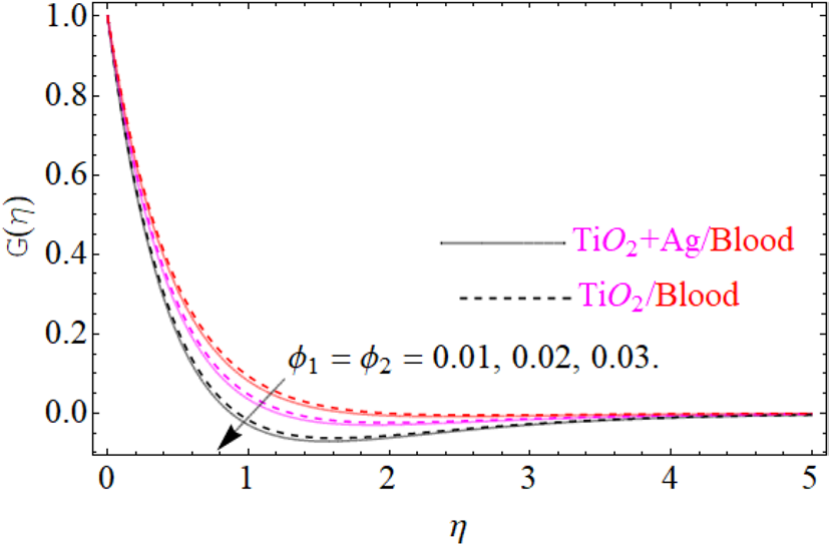
Under the influence of $\left( {\phi_{1} ,\,\,\phi_{2} } \right)$ (nanoparticle volume friction), $G\left( \eta \right)$.

**Figure 8:**
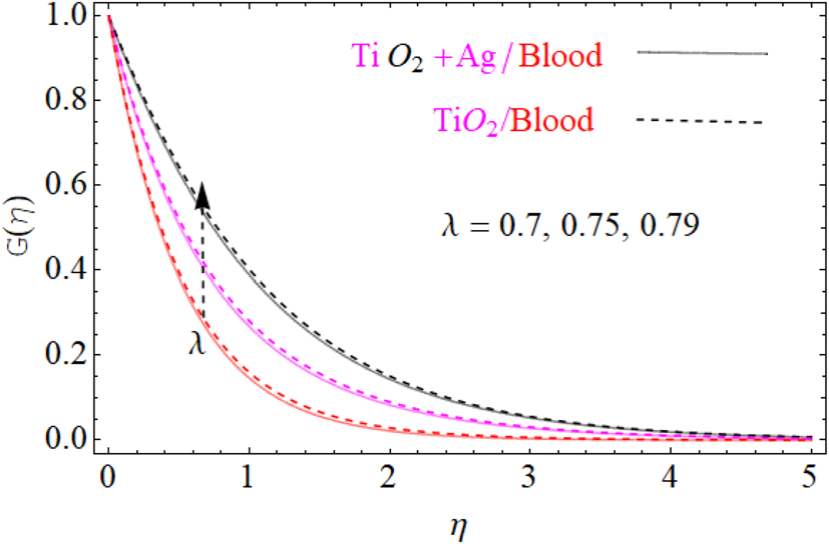
Under the influence of $\lambda$ (rotation parameter), $G\left( \eta \right)$.

**Figure 9:**
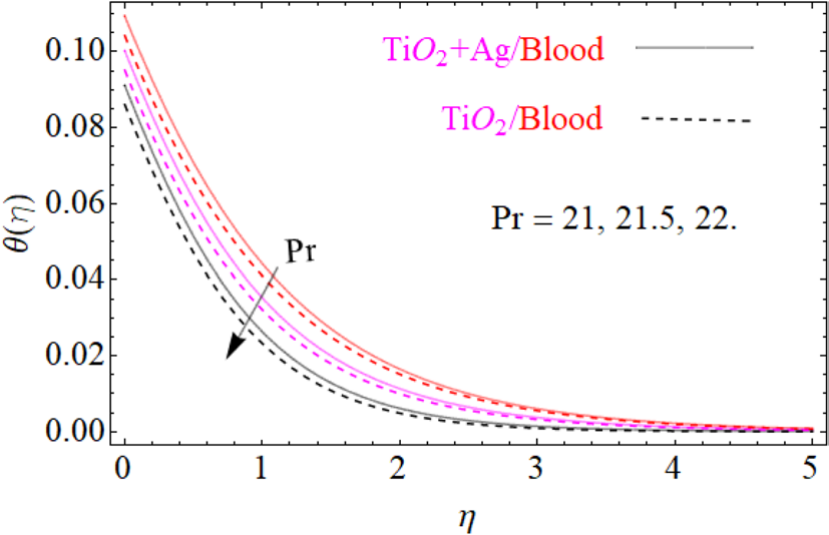
Under the influence of $\Pr$ (Prandtl number), $\theta \left( \eta \right)$.

**Figure 10:**
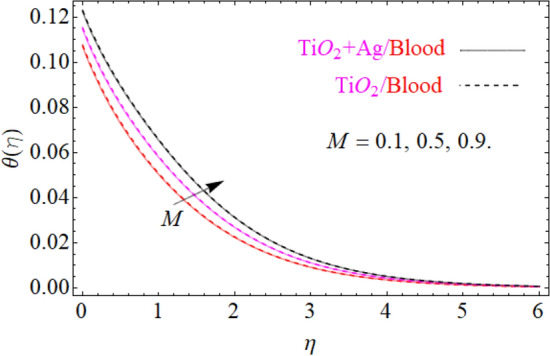
Under the influence of $M$ (Magnetic parameter), $\theta \left( \eta \right)$.

**Figure 11:**
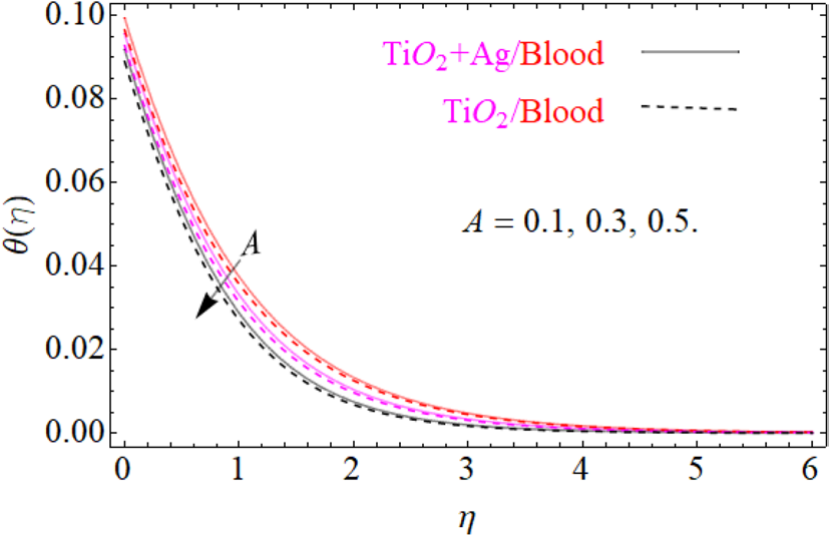
Under the influence of $A$ (unsteadiness parameter), $\theta \left( \eta \right)$.

**Figure 12:**
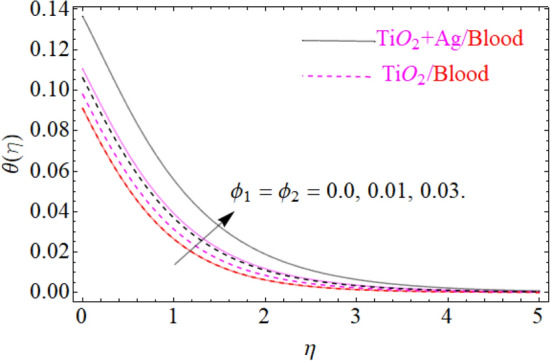
Under the influence of $\left( {\phi_{1} ,\,\,\phi_{2} } \right)$ (nanoparticle concentration parameter), $\theta \left( \eta \right)$.

**Figure 13:**
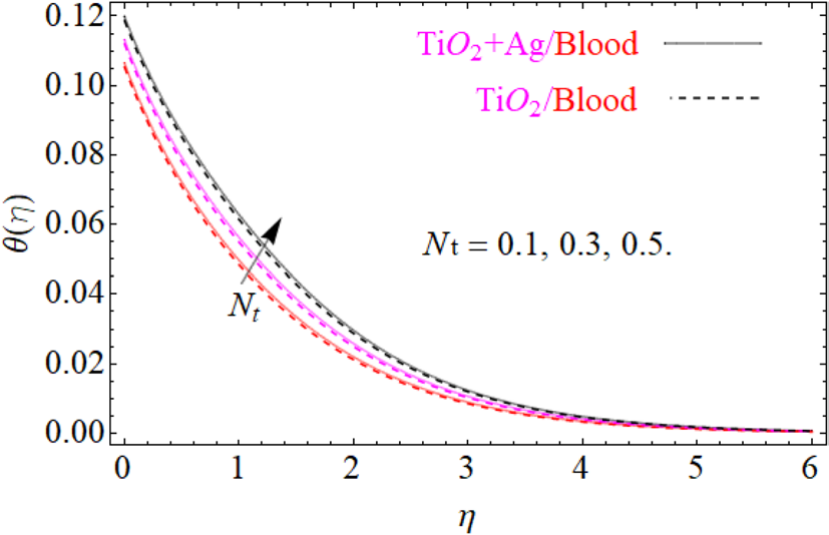
Under the influence of $N_{t}$ (thermophoresis parameter), $\theta \left( \eta \right)$.

**Figure 14:**
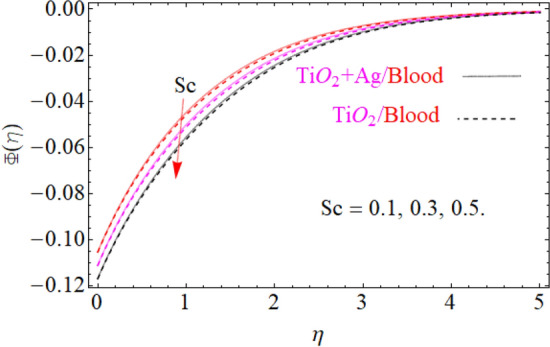
Under the influence of ${\text{Sc}}$ (Schmidt number), $\Phi \left( \eta \right)$.

**Figure 15:**
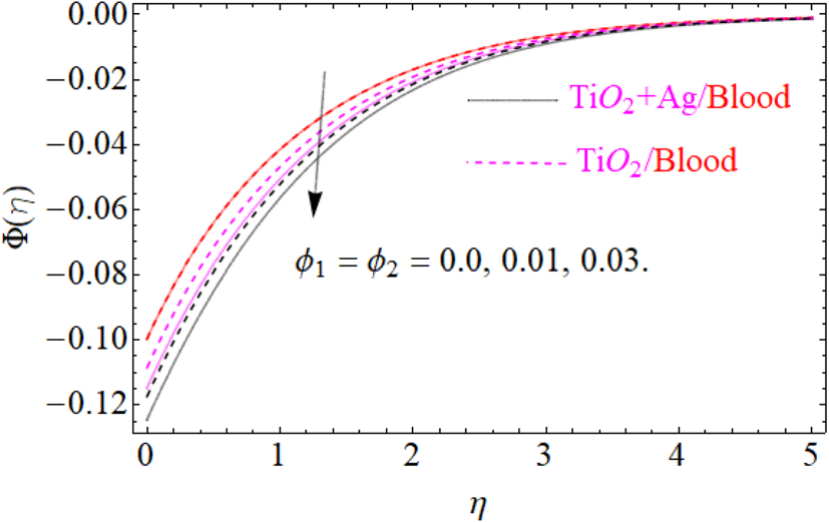
Under the influence of $\left( {\phi_{1} ,\,\,\phi_{2} } \right)$ (nanoparticle concentration parameter), $\Phi \left( \eta \right)$.

**Figure 16:**
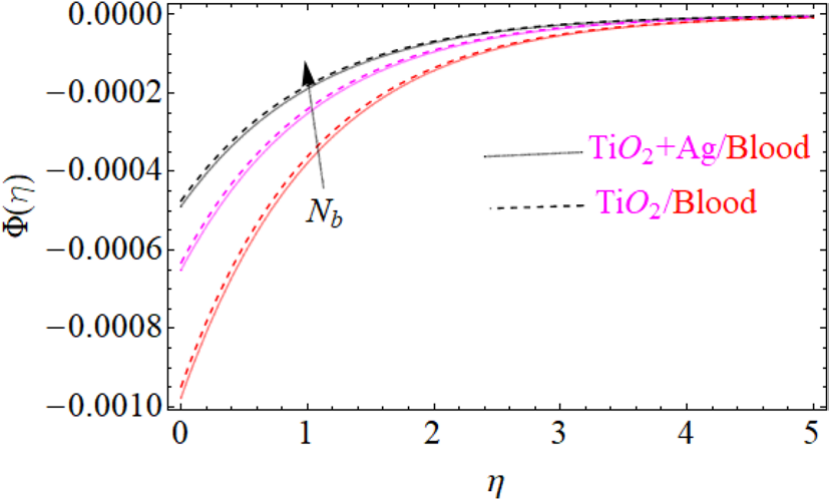
Under the influence of $N_{b}$ (Brownian motion parameter), $\Phi \left( \eta \right)$.

This portion of the current study is concentrated throughout the theoretical analysis of blood base hybrid nanofluid flow around a rotating sphere. From these analyses certain important insights are produced from the graphical configurations of the velocity, temperature, and concentration profile, taking into account various groundbreaking parameters. In the presence of distinct nanoparticles, the whole theoretical analysis is based on comparing ${\text{TiO}}_{2}$/blood and ${\text{TiO}}_{2} + {\text{Ag}}$/blood with the help of the stated problem. Through the aid of some fixed mathematical values of factors like $\phi_{1} = 0.01 - 0.05$ (corresponds to ${\text{TiO}}_{2}$/blood case) and $\phi_{2} = 0.01 - 0.05$ (corresponds to ${\text{TiO}}_{2} + {\text{Ag}}$/bloodcase), $N_{r} = [ - 1,1]$,$N_{b} = [0.2,1.2]$,$N_{t} = [0.2,1.2]$,$A = [0.1,0.8]$,${\text{Sc}} = [0.4,4.0]$,$\lambda = [1.0,10.0]$ , $\lambda^{*} = [1.0,\,10.0]$ these configuration patterns are performed. In the current analysis, two different types of nanoparticles ${\text{TiO}}_{2}$/blood and ${\text{Ag}}$/blood are considered and its detail of thermophysical features of ${\text{TiO}}_{2}$/blood (nanofluid) and ${\text{TiO}}_{2} + {\text{Ag}}$/blood (hybrid nanofluid) are presented in Tables 2 and 3. The thermal-physical characteristics of ${\text{TiO}}_{2}$, ${\text{Ag}}$ and blood at 25 °C are specified in Table 3. The comparison of the published with the present work is displayed in Table 4. Similarly, Table 5 displays the variation of both $C_{f}$ for ${\text{TiO}}_{2}$/blood (nanofluid) and ${\text{TiO}}_{2} + {\text{Ag}}$/blood (hybrid nanofluid). Since $C_{f}$ are directly related to $k^{*} ,\,M,\,\,(\,\phi_{1} ,\,\,\phi_{2} )$. As a consequence, it is dug out that the remarkable nature is investigated for ${\text{TiO}}_{2}$/blood (nanofluid) and ${\text{TiO}}_{2} + {\text{Ag}}$/blood. In addition, it was clear from Table 5 that $C_{f}$ in the case of ${\text{TiO}}_{2} + {\text{Ag}}$/blood (hybrid nanofluid) demonstrated superiority when equated to the ${\text{TiO}}_{2}$/blood (nanofluid). The results of the current analysis for the ${\text{Nu}}$ (heat transfer rate) are exposed in Table 6. It is worth mentioning that ${\text{Nu}}$ is directly related to the $M,\,\,N_{t} ,\,\,(\phi_{1} ,\,\,\phi_{2} )$. So ${\text{Nu}}$ enhances due to the intensification of $M,\,\,N_{t} ,\,\,(\phi_{1} ,\,\,\phi_{2} )$ parameters. The percentage (%) enhancement in the heat transfer rate has been observed with the increment of the nanoparticle volume fractions $\phi_{1} ,\,\,\phi_{2}$. Increase in the $\phi_{1} ,\,\,\phi_{2}$ from 0.01 to 0.02 in case of the ${\text{TiO}}_{2} + {\text{Ag}}$ enhance the thermal conductivity 5.8% and 11.947% respectively. Furthermore, the same value of the nanoparticle volume fraction in case of ${\text{TiO}}_{2}$ enhancing the thermal conductivity 2.576% and 5.197% respectively as shown in Table 6. Also, the convergence of OHAM-BVPh 2.0 package up to 25 orders of approximation is displayed in Tables 7 and 8 for ${\text{TiO}}_{2}$/blood (nanofluid) and ${\text{TiO}}_{2} + {\text{Ag}}$/blood (hybrid nanofluid) respectively. Detailed results of the model problem have been achieved and its corresponding specifications are graphically presented for component of $F^{\prime}\left( \eta \right)$ and $G\left( \eta \right)$ (primary and secondary velocities), $\theta \left( \eta \right)$ temperature field and $\Phi \left( \eta \right)$ nanoparticle concentration filed in Figs. 2, 3, 4, 5, 6, 7, 8, 9, 10, 11, 12, 13, 14, 15, and 16. Figures 2, 3, 4, and 5, we have plotted to describe the physical behavior of ${\text{TiO}}_{2} /blood$ and ${\text{TiO}}_{2} + {\text{Ag}}/blood$ on primary velocities $F^{\prime}\left( \eta \right)$ for variations in several variables present in the equation of motion. Such as $k^{*} ,\,\phi_{1} ,\,\phi_{2} ,\,M$ and $\lambda^{*}$ on flow is investigated graphically. Figure 2 depicts a particular image of the $F^{\prime}\left( \eta \right)$ (primary velocity) profile for various values of $k^{*}$(couple stress variable). It is predicted from the figure that $F^{\prime}\left( \eta \right)$ (primary velocity) for both cases of fluid (${\text{TiO}}_{2} /blood$ and ${\text{TiO}}_{2} + {\text{Ag}}/blood$) declines as enhancing the value of $k^{*}$. The explanation is so clear. By strengthening $k^{*}$ variable slow down the motion of both fluid (${\text{TiO}}_{2} /blood$ and ${\text{TiO}}_{2} + {\text{Ag}}/blood$) due to an increasing the drag force which is equal to an obvious reduction in the fluid viscosity. From such parabolic images, one should observe that the velocity of ${\text{TiO}}_{2} + {\text{Ag}}/blood$ case gives frequent decline relative to the case of ${\text{TiO}}_{2} /blood$ nanofluid. The influences of the first $TiO_{2}$ and the second $Ag$ nanoparticle volume fraction $\left( {\phi_{1} ,\,\phi_{2} } \right)$ on the primary velocity field $F^{\prime}\left( \eta \right)$ is graphically presented in Fig. 3. The primary velocity field $F^{\prime}\left( \eta \right)$ is clearly seen to be remarkably declined against the enhancing value of the nanoparticle volume fraction $TiO_{2}$ and $Ag$ $\left( {\phi_{1} ,\,\phi_{2} } \right)$ of fluids. Physically, the higher values of the nanoparticle volume fraction of ${\text{TiO}}_{2} /blood$ and ${\text{TiO}}_{2} + {\text{Ag}}/blood$ causes the thinning behavior of the momentum boundary layer. This should also be observed that the sharp decline in nanofluid ${\text{TiO}}_{2}$ velocity is lower than that for hybrids nanofluid $\left( {{\text{TiO}}_{2} + {\text{Ag}}/blood} \right)$. The effect of the $M$(magnetic parameter) on the ${\text{TiO}}_{2} + {\text{Ag}}/blood$ and ${\text{TiO}}_{2} + {\text{Ag}}/blood$ $F^{\prime}\left( \eta \right)$ primary velocities is investigated and the fundamental physics is visually illustrated in Fig. 4. Figure 4 indicates that when the $M$(magnetic field) is raised, then the velocity field of ${\text{TiO}}_{2} /blood$ and ${\text{TiO}}_{2} + {\text{Ag}}/blood$ fluid sluggish. This declining influence on the $F^{\prime}\left( \eta \right)$ velocity of the ${\text{TiO}}_{2} + {\text{Ag}}/blood$ is more prominent than ${\text{TiO}}_{2} /blood$. Such decline state of velocities happens owing to the production of resistant type force identified as Lorentz force. The strength of such force enhances with the rising strength of $M$ which counteracts the motion of fluid in boundary film and drop the viscosity of boundary film. The effect of $\lambda^{*}$(mixed convection parameters) on ${\text{TiO}}_{2} /blood$ and ${\text{TiO}}_{2} + {\text{Ag}}/blood$ fluid of $F^{\prime}\left( \eta \right)$ primary velocity is demonstrated in Fig. 5. In order to increase the $\lambda^{*}$(mixed convection parameters) the $F^{\prime}\left( \eta \right)$ primary velocity is significantly accelerated. A $F^{\prime}\left( \eta \right)$ velocity enhancing is reported, through a greater magnitude of the $\lambda^{*}$ which could be due to the high buoyancy force. The supporting buoyancy force works as a desirable pressure gradient and significantly speeds up $F^{\prime}\left( \eta \right)$. This accelerating influence on $F^{\prime}\left( \eta \right)$ of the ${\text{TiO}}_{2} + {\text{Ag}}$/blood is more prominent than that of the nanofluid. Figures 6, 7, and 8, we have plotted to describe the physical behavior of ${\text{TiO}}_{2} /blood$ and ${\text{TiO}}_{2} + {\text{Ag}}$/blood on the secondary velocity $G\left( \eta \right)$ field for variations in several of model equations. The physical nature of these model factors $\,\phi_{1} ,\,\phi_{2} ,\,M$ and $\lambda$ on flow is graphically studied. The influence of the $M$(magnetic field) on the both (${\text{TiO}}_{2} /blood$ and ${\text{TiO}}_{2} + {\text{Ag}}$/blood) nanofluid $G\left( \eta \right)$ secondary velocities is also examined and the essential physics is visually demonstrating in Fig. 6. Figure 6 specifies that when the magnitude of $M$ is more elevated than the $G\left( \eta \right)$ velocity field of ${\text{TiO}}_{2} /blood$ and ${\text{TiO}}_{2} + {\text{Ag}}/blood$ fluid slow down. Such diminishing effect on the $G\left( \eta \right)$ of the ${\text{TiO}}_{2} + {\text{Ag}}/blood$ is much more prominent than the ${\text{TiO}}_{2} /blood$. Such decay condition of $G\left( \eta \right)$ velocities happen due to the creation of protected type force identified as Lorentz force. The power of such force improves with the growing strength of $M$ which offsets the speed of both fluids within boundary layer and drop the thickness of the boundary layer. The $G\left( \eta \right)$ vs first ${\text{TiO}}_{2}$ and second ${\text{Ag}}$ nanoparticle volume fraction $\left( {\phi_{1} ,\,\phi_{2} } \right)$ behaviors are seen in Fig. 7, that indicates that $G\left( \eta \right)$ of both (${\text{TiO}}_{2} /blood$ and ${\text{TiO}}_{2} + {\text{Ag}}$/blood) decreases as the $\left( {\phi_{1} ,\,\phi_{2} } \right)$ increases. Substantially, the higher values of both $\left( {\phi_{1} ,\,\phi_{2} } \right)$ of nanofluid and hybrid nanofluid cause the thinning behavior of the momentum boundary layer. This should also be observed that the sharp decline in nanofluid ${\text{TiO}}_{2}$ velocity is lower than that for hybrids nanofluid $\left( {{\text{TiO}}_{2} + {\text{Ag}}/blood} \right)$. The effect of the $\lambda$ (rotation parameter) of ${\text{TiO}}_{2} /blood$ and ${\text{TiO}}_{2} + {\text{Ag}}/blood$ fluid on $G\left( \eta \right)$ secondary velocity components is seen in Fig. 8. In order to upsurge the magnitude of $\lambda$, the $G\left( \eta \right)$ secondary velocity components are significantly accelerated. It is clear from the diagram that the rotation becomes much more intense by increasing the λ, and the $G\left( \eta \right)$ secondary momentum helps more through the swirl effect that retards the secondary movement $G\left( \eta \right)$. From this parabolic plot, one should perceive that the velocity of ${\text{TiO}}_{2} + {\text{Ag}}/blood$ case gives frequent increase relative to the case of ${\text{TiO}}_{2} /blood$ nanofluid. The Figs. 9, 10, 11, 12, and 13, afterward demonstrating the effect of $\Pr ,\,\,M,\,A,\,\left( {\phi_{1} ,\,\phi_{2} } \right)$ and $N_{t}$ on $\theta (\eta )$ for both (${\text{TiO}}_{2} /blood$ and ${\text{TiO}}_{2} + {\text{Ag}}$/blood). To scrutinize the trend of $\Pr$ on $\theta \left( \eta \right)$, Fig. 9 is plotted for both cases of (${\text{TiO}}_{2} /blood$ and ${\text{TiO}}_{2} + {\text{Ag}}$/blood). It is evident from the plot that the $\theta \left( \eta \right)$ field shows the diminishing role for $\Pr$. Consequently, it is defensible owing to the basic reason that thermal conductivity of the fluid relative lesser with an intensified magnitude of $\Pr$ and thus reducing the temperature of both (${\text{TiO}}_{2} /blood$ and ${\text{TiO}}_{2} + {\text{Ag}}$/blood). It's also reported herein that in the scenario of ${\text{TiO}}_{2} + {\text{Ag}}/blood$ the $\theta \left( \eta \right)$ temperature field reached its maximum value as compared to the ${\text{TiO}}_{2} /blood$ nanofluid case. The variance in $M$ (magnetic parameter) on temperature $\theta \left( \eta \right)$ profile is depicted in Fig. 10 for both (${\text{TiO}}_{2} /blood$ and ${\text{TiO}}_{2} + {\text{Ag}}$/blood). Form this drawing it is detected that $\theta \left( \eta \right)$ and thermal layer thickness enhances via growing magnitude of magnetic parameter $M$. The magnetic parameter and density of (${\text{TiO}}_{2} /blood$ and ${\text{TiO}}_{2} + {\text{Ag}}$/blood) are inversely related with each other. Hence, the strengthening value of $M$ shrinking the density and as a consequence the thermal nature of fluid upsurges. From this outline, one can observe that the temperature for ${\text{TiO}}_{2} + {\text{Ag}}/blood$ case tends to decrease more quickly relative to the case of ${\text{TiO}}_{2} /blood$. The effect of the $A$ (unsteadiness parameter) on both (${\text{TiO}}_{2} /blood$ and ${\text{TiO}}_{2} + {\text{Ag}}$/blood), $\theta \left( \eta \right)$ temperature profiles is revealed in Fig. 11. This can be seen from a plot that are rising the magnitude of $A$ leads to a reduction in the temperature field. Physically, with increasing values of $A$, the $\theta \left( \eta \right)$ field is reduced, indicating that the thermal boundary surfaces are diminished with the growing magnitude of $A$. Therefore, the boundary layer is cooler, so additional temperature and nanoparticles are moved to the surface of the sphere (wall) with a growing magnitude of $A$. It's also described herein that in the scenario of ${\text{TiO}}_{2} + {\text{Ag}}/blood$ the $\theta \left( \eta \right)$ temperature field reached its maximum value as compared to the ${\text{TiO}}_{2} /blood$ nanofluid case. The effect of $\left( {\phi_{1} ,\,\phi_{2} } \right)$ on $\theta (\eta )$ is also studied and the basic reason is obviously portrayed in Fig. 12. From the drawing it is observed that the $\theta (\eta )$ is enhanced for the increase in the volume concentration of both nanoparticles ${\text{TiO}}_{2} /blood$ and hybrid ${\text{TiO}}_{2} + {\text{Ag}}/blood$, which can be attributed to the collisions between the suspended ${\text{TiO}}_{2} ,\,\,{\text{Ag}}$ nanoparticles. $\left( {\phi_{1} ,\,\phi_{2} } \right)$ is basically link to both ${\text{TiO}}_{2} ,\,\,{\text{Ag}}$ nanoparticles, therefore, the effect of both nanoparticles on $\theta (\eta )$ is observed. Mostly, the stimulation of thermal boundary film viscosity to $\left( {\phi_{1} ,\,\phi_{2} } \right)$ is linked to upgraded thermal conduction of both (${\text{TiO}}_{2} /blood$ and ${\text{TiO}}_{2} + {\text{Ag}}/blood$). In fact, superior amount of thermal conduction is reinforced by improved thermal diffusivity. Actually, $\theta \left( \eta \right)$ field is directly related to both $\left( {\phi_{1} ,\,\phi_{2} } \right)$ in case of contraction and in this scenario, it displays extra inconsistency closed to the wall of the sphere. Due to evolving $N_{t}$ (thermophoresis parameter), this paragraph is dedicated to capturing the variation in the $\theta \left( \eta \right)$ temperature profile for both nanoparticles ${\text{TiO}}_{2} /blood$ and hybrid ${\text{TiO}}_{2} + {\text{Ag}}/blood$. Figure 11 is depicted in order to observe the way that $\theta \left( \eta \right)$ temperature is affected. Figure 13 illustrates that the magnitude of both (${\text{TiO}}_{2} /blood$ and ${\text{TiO}}_{2} + {\text{Ag}}/blood$) temperature grows by enhancing $N_{t}$. Basically, it is how the thermophoresis force allows the particles to shift from the hot zone to the cold area, improves as $N_{t}$ raises and the thermophoresis force improves which upgrades the magnitude of $\theta \left( \eta \right)$ temperature field magnitude of nanofluids ${\text{TiO}}_{2} /blood$ and hybrid nanofluids ${\text{TiO}}_{2} + {\text{Ag}}/blood$. Figures 14, 15, and 16, we have designed to define the physical characteristics of ${\text{TiO}}_{2} /blood$ and ${\text{TiO}}_{2} + {\text{Ag}}$/blood on concentration profile $\Phi \left( \eta \right)$ for variations in several variable present in the model equations. Such as ${\text{Sc}},\,\,(\phi_{1} ,\,\phi_{2} )$ and $N_{b}$ on concentration profile $\Phi \left( \eta \right)$ is investigated graphically. The consequence of the ${\text{Sc}}$ (Schmidt Number) on $\Phi \left( \eta \right)$(concentration) profiles is portrayed in Fig. 14 for the case of (${\text{TiO}}_{2} /blood$ and ${\text{TiO}}_{2} + {\text{Ag}}/blood$). The $\Phi \left( \eta \right)$ magnitude is reduced slightly substantially with rising values of ${\text{Sc}}$. Basically, the relation of momentum diffusivity to mass (nanoparticle) diffusivity is called ${\text{Sc}}$. For ${\text{Sc}} < 1$, diffusivity in mass dominates diffusivity in momentum, and vice versa for ${\text{Sc}} > 1$. Hence, with higher ${\text{Sc}}$, concentration boundary-layer density for both nanoparticles ${\text{TiO}}_{2} ,\,\,{\text{Ag}}$ is substantially enhanced, while the thermal boundary-layer density is marginally reduced. In order to see the influence of volumetric fractions of nanomaterials $(\phi_{1} ,\,\phi_{2} )$ on the concentration profile $\Phi \left( \eta \right)$, Fig. 15 has been plotted for both (${\text{TiO}}_{2} /blood$ and ${\text{TiO}}_{2} + {\text{Ag}}$/blood). It is noticeable that the decline in solute concentration was perceived by growing the volumetric fraction of nano additives $(\phi_{1} ,\,\phi_{2} )$. In addition, lower values for ${\text{TiO}}_{2} + {\text{Ag}}$/blood, hybrid nanofluid were observed in the $\Phi \left( \eta \right)$ profile when equated to ${\text{TiO}}_{2} /blood$ nanofluid. The effect of the $\Phi \left( \eta \right)$ concentration profile of the $N_{b}$ (Brownian motion parameter) is displayed in Fig. 16 for the case of (${\text{TiO}}_{2} /blood$ and ${\text{TiO}}_{2} + {\text{Ag}}$/blood). An improvement to $N_{b}$ lead to accelerates $\Phi \left( \eta \right)$ as seen in Figure. Physically, it is attributable to disperse through the (${\text{TiO}}_{2} ,{\text{Ag}}$) particles, fluid improvements with the maximization of $N_{b}$. It is therefore understood that in the case of ${\text{TiO}}_{2} + {\text{Ag}}$/blood, hybrid nanofluid, as can be seen in the figure the temperature gets its highest values as compared with ${\text{TiO}}_{2} /blood$ nanofluid.

**Table 3:** Various thermo-physical characters of blood, Silver and Titania^36^.

Thermo-physical Prop.	Size (nm)	$c_{p} \;({\text{J}}/{\text{kgK}})$	$\rho \;\left( {{\text{kg}}/{\text{m}}^{3} } \right)$	$k\;({\text{W}}/{\text{mK}})$
Silver:${\text{Ag}}$	2–5	235	10,500	429
Titania: ${\text{TiO}}_{2}$ (Titanium dioxide)	50	686.2	4250	8.954
Pure blood	–	3594	1063	0.492

**Table 4:** Comparison between the present results and Ref for the velocity gradient at different values of ${\text{Re}}$ for $\phi_{1} = \phi_{2} = k^{*} = 0,\lambda = 0.2,N_{t} = N_{b} = {\text{Sc}} = 0.4,\,\Pr = 21,{\text{Nr}} = 0.5$.

$M$	$F^{\prime\prime}\left( 0 \right)$	$G^{\prime}\left( 0 \right)$	$F^{\prime\prime}\left( 0 \right)$	$G^{\prime}\left( 0 \right)$
	Ref^24^ results	Ref^24^ results	Current result	Current result
0.1	1.1299	− 0.634632	1.12994579	− 0.6346328456
0.3	1.17448	− 0.663496	1.174483467	− 0.6634967345
0.5	1.21899	− 0.692221	1.218992356	− 0.6922215234
0.7	1.26345	− 0.720806	1.263452314	− 0.7208063421

**Table 5:** Evaluation of the Skin friction coefficients $F^{\prime\prime}(0),G^{\prime}(0)$ for selected values of $A = 1.6,\phi_{1} = 0.04$.

$k^{*}$	$M$	$\phi_{1} ,\phi_{2}$	$$\begin{aligned} & F^{\prime\prime}(0) \\ & {\text{TiO}}_{2} + {\text{Ag}} \\ \end{aligned}$$	$$\begin{aligned} & F^{\prime\prime}(0) \\ & {\text{TiO}}_{2} \\ \end{aligned}$$	$$\begin{aligned} & G^{\prime}(0) \\ & {\text{TiO}}_{2} + {\text{Ag}} \\ \end{aligned}$$	$$\begin{aligned} & G^{\prime}(0) \\ & {\text{TiO}}_{2} \\ \end{aligned}$$
0.1	0.1	0.01	2.32541	2.12971	1.3469128	1.335724
0.3			2.32750	2.14382	1.3478239	1.336736
0.5			2.37110	2.16261	1.3488348	1.337748
0.1	0.2		2.87276	2.59942	1.3484724	1.339258
	0.3		2.89991	2.63724	1.3585232	1.341457
0.1	0.1	0.00	2.33763	2.11645	1.3458017	1.342372
		0.02	3.48962	3.27468	1.3512781	1.351278
		0.04	3.65905	3.38579	1.3573238	1.357323

**Table 6:** Evaluation of the heat transfer rate for selected values of $\Pr = 21,A = 0.9,$ using the % formula $\% \;{\text{Increase}} = \frac{{{\text{With}}\,{\text{Nanoparticle}}}}{{{\text{Without}}\,{\text{Nanoparticle}}}} \times 100 = {\text{Result,}}\;{\text{Result}} - {100} = \% \,{\text{enhancment}}$.

$M$	$Nt$	$\phi_{1} = \phi_{2}$	$$\begin{aligned} & \theta^{\prime}(0) \\ & {\text{TiO}}_{2} + {\text{Ag}} \\ \end{aligned}$$	$$\begin{aligned} & \theta^{\prime}(0) \\ & {\text{TiO}}_{2} \\ \end{aligned}$$
1	0.1	0.01	0.05123	0.037077
1.5	0.1		0.05934	0.037823
2	0.1		0.06745	0.038039
2	0.2		0.06956	0.038564
2	0.3		0.07376	0.038917
2	0.4		0.07959	0.039021
2	0.4		0.09502	0.039346
		0.0	0.0424654	0.0492
		0.01	0.0449406 (5.8% Increase)	0.0504676 (2.576% Increase)
		0.02	0.0475391 (11.947% Increase)	0.0517571 (5.197% Increase)

**Table 7:** Convergence of OHAM for ${\text{TiO}}_{2} + {\text{Ag}} + Blood$.

$m$	$\varepsilon_{m}^{f} \,{\text{TiO}}_{2} + {\text{Ag}} + Blood$	$\varepsilon_{m}^{\theta } {\text{TiO}}_{2} + {\text{Ag}} + Blood$
_5_	$1.16438 \times 10^{ - 1}$	$1.16775 \times 10^{ - 3}$
_10_	$2.14094 \times 10^{ - 2}$	$3.18738 \times 10^{ - 5}$
_15_	$0.209443 \times 10^{ - 3}$	$0.07298 \times 10^{ - 7}$
_20_	$1.37298 \times 10^{ - 5}$	$1.54131 \times 10^{ - 8}$
_25_	$3.95787 \times 10^{ - 7}$	$2.14423 \times 10^{ - 9}$

**Table 8:** Convergence of OHAM for ${\text{TiO}}_{2} + Blood$.

$m$	$\varepsilon_{m}^{f} \,{\text{TiO}}_{2} + Blood$	$\varepsilon_{m}^{\theta } {\text{TiO}}_{2} + Blood$
_5_	$0.07991 \times 10^{ - 1}$	$0.18574 \times 10^{ - 1}$
_10_	$0.65266 \times 10^{ - 3}$	$.0759 \times 10^{ - 2}$
_15_	$1.11383 \times 10^{ - 5}$	$.0759 \times 10^{ - 5}$
_20_	$0.4616 \times 10^{ - 6}$	$0.32721 \times 10^{ - 7}$
_25_	$1.133 \times 10^{ - 9}$	$0.106632 \times 10^{ - 9}$

## Concluding remarks

The circulation of the ${\text{TiO}}_{2} + {\text{Ag}}$/blood, hybrid nanofluid was examined around a rotating sphere under the action of a uniform applied magnetic field. The velocity, temperature along with concentration distribution, was also scientifically and systematically analyzed by taking the effects of $N_{b}$,$N_{t}$ and $(\phi_{1} ,\,\phi_{2} )$ into consideration. The main results of this analysis were the following:

- The $F^{\prime}\left( \eta \right)$ component of primary velocity, declined with the boosting value of $(\phi_{1} ,\,\phi_{2} )$ and $M$, while an accelerating behavior was perceived via enhancing value of $\lambda^{*}$.
- Also, the $G\left( \eta \right)$ secondary velocity component, boosted with the enhancing value of $\lambda$, where declinatory conduct was found through increasing values of $(\phi_{1} ,\,\phi_{2} )$ and $M$.
- In contrast to ${\text{TiO}}_{2}$ nanofluid, the ${\text{TiO}}_{2} + {\text{Ag}}$/blood (hybrid nanofluid) demonstrated a weak velocity profile of $F^{\prime}\left( \eta \right)$ and $G\left( \eta \right)$.
- For the growing values of $(\phi_{1} ,\,\phi_{2} ),\,N_{t}$ and $M$, temperature elevation was chronicled. In those scenarios, the ${\text{TiO}}_{2} + {\text{Ag}}$/blood (hybrid nanofluid) exhibited the greatest temperature as equated to ${\text{TiO}}_{2}$ nanofluid.
- With the increasing value of $N_{b}$, an increase in the $\Phi \left( \eta \right)$ was observed. However, an opposite pattern was reported in increasing $\left( {\phi_{1} ,\,\phi_{2} } \right)\,\,{\text{and}}\,\,{\text{Sc}}$.
- ${\text{Nu}}\,\,{\text{and}}\,\,C_{f}$ demonstrated considerable superiority in the case of ${\text{TiO}}_{2} + {\text{Ag}}$/blood (hybrid nanofluid) as equated to ${\text{TiO}}_{2}$ nanofluid.
- There was considerable realistic relevance to the current study, particularly in the field of biomedical and chemical industries.
- The output shows that the increase in the $\phi_{1} ,\,\,\phi_{2}$ from 0.01 to 0.02 in case of the ${\text{TiO}}_{2} + {\text{Ag}}$ enhance the thermal conductivity 5.8% and 11.947% and for the same value of the nanoparticle volume fraction in case of ${\text{TiO}}_{2}$ enhancing the thermal conductivity 2.576% and 5.197%.

## Data Availability

The data that support the findings of this study are available from the corresponding author upon reasonable request.

## List of symbols

$$u$$, $$v$$$$w$$: Velocities components $$\left( {{\text{ms}}^{ - 1} } \right)$$
$B_{0}$: Magnetic field strength $\left( {{\text{Nm}}\;{\text{A}}^{ - 1} } \right)$
$F,G$: Dimensional velocity profiles
$$T$$: Fluid temperature $$\left( {\text{K}} \right)$$
$$T_{w}$$: Surface temperature $$\left( {\text{K}} \right)$$
$T_{\infty }$: Free surface temperature $\left( {\text{K}} \right)$
$$C_{w}$$: Surface concentration
$$M$$: Magnetic parameter
$D_{B}$: Coefficients of Brownian movement
$D_{T}$: Diffusion coefficients of thermophoresis
$${\text{Pr}}$$: Prandtl number
${\text{Re}}$: Local Reynolds number
$${\text{Ec}}$$: Eckert number
$${\text{Gr}}$$: Grashof number
$${\text{{\rm N}u}}$$: Nusselt number
${\text{C}}_{f}$: Skin friction coefficient
$k^{*}$: Couple stress parameter
$$B_{i}$$: Biot number
$\left( {C_{p} } \right)_{f}$: Specific heat of base fluid $\left( {{\text{J}}/{\text{kgK}}} \right)$
$$k_{nf}$$: Thermal conductivity $$\left( {{\text{Wm}}^{ - 1} {\text{K}}^{ - 1} } \right)$$

## Greek symbols

$\mu_{nf}$: Dynamic viscosity of nanofluid $\left( {{\text{mPa}}} \right)$
$\mu_{f}$: Dynamic viscosity of base fluid $\left( {{\text{mPa}}} \right)$
$$\rho_{nf}$$: Nanofluid density $$\left( {{\text{kg}}\;{\text{m}}^{ - 3} } \right)$$
$\rho_{f}$: Base fluid density $\left( {{\text{kg}}\;{\text{m}}^{ - 3} } \right)$
$$\eta$$: Similarity variable
$\phi_{1}$ and $\phi_{2}$: Volume fraction of ${\text{TiO}}_{2}$ and ${\text{Ag}}$ nanoparticles
$\Phi$: Dimensional concentration profile
$\theta$: Dimensional heat profile
$\sigma_{nf}$: Electrical conductivity of nanofluid
$$\lambda$$: Rotation parameter
$\lambda^{*}$: Mixed convection parameter
$\varepsilon_{m}^{t}$: The total residual error

## Subscripts

nf: Nanofluid
f: Base fluid
hnf: Hybrid nanofluid

## Abbreviations

OHAM: Optimal homotopy analysis method
MHD: Magneto-hydrodynamics
CNTs: Carbon nanotubes
